# Integrated analysis of lncRNA and mRNA expression in rainbow trout families showing variation in muscle growth and fillet quality traits

**DOI:** 10.1038/s41598-018-30655-8

**Published:** 2018-08-14

**Authors:** Ali Ali, Rafet Al-Tobasei, Brett Kenney, Timothy D. Leeds, Mohamed Salem

**Affiliations:** 1 Department of Biology and Molecular Biosciences Program, Middle Tennessee State University, Murfreesboro, TN 37132 USA; 2 Computational Science Program, Middle Tennessee State University, Murfreesboro, TN 37132 USA; 3 Department of Biostatistics, University of Alabama at Birmingham, Birmingham, AL 35294-0022 USA; 4 Division of Animal and Nutritional Science, West Virginia University, Morgantown, WV 26506-6108 USA; 5 The National Center for Cool and Cold Water Aquaculture, USDA Agricultural Research Service, Kearneysville, WV 25430 USA

## Abstract

Muscle yield and quality traits are important for the aquaculture industry and consumers. Genetic selection for these traits is difficult because they are polygenic and result from multifactorial interactions. To study the genetic architecture of these traits, phenotypic characterization of whole body weight (WBW), muscle yield, fat content, shear force and whiteness were measured in ~500 fish representing 98 families from a growth-selected line. RNA-Seq was used to sequence the muscle transcriptome of different families exhibiting divergent phenotypes for each trait. We have identified 240 and 1,280 differentially expressed (DE) protein-coding genes and long noncoding RNAs (lncRNAs), respectively, in fish families exhibiting contrasting phenotypes. Expression of many DE lncRNAs (n = 229) was positively correlated with overlapping, neighboring or distantly located protein-coding genes (n = 1,030), resulting in 3,392 interactions. Three DE antisense lncRNAs were co-expressed with sense genes known to impact muscle quality traits. Forty-four DE lncRNAs had potential sponge functions to miRNAs that affect muscle quality traits. This study (1) defines muscle quality associated protein-coding and noncoding genes and (2) provides insight into non-coding RNAs involvement in regulating growth and fillet quality traits in rainbow trout.

## Introduction

Aquaculture is the fastest growing agribusiness with potential to improve food security and expand economic opportunities worldwide^1^. Regarding the US Trout Industry, a key challenge for this industry is expansion to meet increasing consumer demand^2^. Additional effort should focus on development of genetically improved strains to achieve fast/efficient production of fish with high fillet yields and quality^2^.

Fillet is the most nutritional and economically important part of the fish; it is high in protein and, depending on the species, is relatively low in fat^3^. Muscle yield and flesh quality greatly affect fish processing profitability^4^. Several variables impact salmonid muscle yield such as harvest weight and age endpoint, animal nutrition^5,6^, sexual status^7^, and genetic factors^8^. Fillet quality attributes, such as fat content, color and texture, are the primary determinants of consumer acceptability^9^. Recently, genetic selection was introduced in rainbow trout to improve flesh quality^10,11^. Selection on fat content affected color and fillet texture^12^, and this approach improved feed conversion ratio (FCR) and protein-retention efficiency^13^ in rainbow trout. Moreover, selection on harvest weight can improve growth rate^14^ and flesh color, in addition to reducing production cost^15^. Selective breeding improves heritable traits through the existing genetic variation between individuals/families. If flesh qualities are incorporated in selection programs, family selection can result in progress toward enhancing these traits^16^. Gjedrem’s studies showed advances in body weight using selection over three generations^17^. A family-based selection line for growth was established in 2002 at the USDA National Center of Cool and Cold Water Aquaculture (NCCCWA); five generations of selection yielded a genetic gain of approximately 10% in harvest body weight per generation^18^. Identifying markers that are associated with muscle growth and quality will improve these traits through selective breeding. In this study, we examined variation of muscle yield and quality traits in hatch year 2010 (third-generation families) of the NCCCWA growth-selected line. In addition, we profiled transcriptome expression of fish families showing contrasting phenotypes in whole body weight (WBW), muscle yield, muscle fat content, shear force, and whiteness index.

The ENCODE project showed that only 1–2% of the human genome encodes for proteins; a major category of the transcribed part represents noncoding RNAs that include miRNAs and lncRNAs. Expression of lncRNAs is regulated according to physiological demands of the cell, suggesting a role for lncRNAs as key regulators of gene expression. Recent studies have demonstrated that lncRNAs contribute to regulation of various cellular processes including cell cycle, apoptosis, differentiation and development, diseases and immunity^19–28^. Genome-wide studies identified lncRNAs exhibiting differential expression during skeletal muscle differentiation^29–31^. Some lncRNAs were experimentally validated as participants in regulation of myogenesis including H19, malat1, MyoD upstream ncRNA (MUNC), lncMyoD, developmental pluripotency-associated 2 (Dppa2), Upstream binding Muscle lncRNA (DUM), and Linc-MD1^32–35^.

LncRNA molecular sponging, or sequestration of miRNA, has been reported recently as an important mode of action of many lncRNAs including H19^33^, Linc-MD1 and malat1^32,36^. H19 harbors miR-675^33^ and Let-7 family^37^ target sites permitting participation in regulation of myogenesis. Linc-MD1 and malat1 have been reported as miR- 133 sponges. miR- 133 regulates mRNA abundance of important myogenic transcription factors such as serum response factor (SRF) and myocyte enhancer factor 2 C (Mef2C)^32,36^. However, sponging is not the only mechanism of action of lncRNAs in regulating skeletal muscle differentiation. LncRNAs can act in *cis-* or *trans-* configurations to regulate neighboring or distant genes. For example, lncMyoD, located away from MyoD locus, binds to IGF2-mRNA-binding protein 2 (IMP2) that controls genes promoting cell cycle arrest. Knockdown of lncMyoD resulted in increased activity of IMP2 and impaired myogenesis^38^. MyoD upstream ncRNA (MUNC) is 5 kb upstream of MyoD locus and regulates the activity of the latter by enhancing the 5 kb region (distal regulatory region)^34^. Dppa2 Upstream Binding Muscle lncRNA (DUM), located near Dppa2 gene, has been reported as a regulator of myogenesis by recruiting DNA methyl-transferase (Dnmt) family members to repress neighboring genes^39^.

Previous studies suggested involvement of miRNAs, transcription factors and other regulatory molecules in controlling muscle growth and fillet quality traits^40–42^. However, role of lncRNA in regulating these traits is still not well understood. Therefore, the objective of this study was to identify the interplay between lncRNAs and protein-coding genes in families with contrasting muscle growth and fillet quality phenotypes. We identified hundreds of protein-coding genes that were co-expressed with DE lncRNAs. Moreover, we found lncRNAs acting as natural sponges for microRNAs, and searched for common miRNA target sites in co-expressed protein-coding and lncRNA genes. We identified co-expressed protein-coding and lncRNA genes harboring binding sites to *cis* regulatory elements of transcription factors involved in myogenesis. This study improves our understanding of the role of protein-coding genes and lncRNAs in (1) muscle growth, and (2) mechanisms underlying variations in phenotypes studied. Additionally, this work will help identify genetic markers for genomic selection in development of improved germplasm for aquaculture.

## Material and Methods

### Ethics statement

Fish were maintained at the USDA National Center of Cool and Cold Water Aquaculture (NCCCWA) and all experimental protocols and animal procedures were approved and carried out in accordance with the guidelines of NCCCWA Institutional Animal Care and Use Committee Protocols #053 and #076.

### Tissue sampling and phenotypic data collection

Fish population and sampling were done as we previously described^43^. Briefly, phenotypic data and muscle samples were collected from ~500 female fish representing 98 families (~5 fish/family) from the growth-selected line at NCCCWA (year class 2010)^14,43^. Full-sib families were produced from single-sire × single-dam matings over a 6-week period. Eggs were reared in spring water, and incubation temperature was manipulated between 7 and 13 °C so that all families hatched within a 3-week period. Each family was reared at ambient water temperature (~12.5 °C) in a separate 200-L tank to retain pedigree information and were fed a commercial fishmeal-based diet (Zeigler Bros Inc., Gardners, PA) using a programmable robotic feeding system (Arvotec, Huutokoski, Finland). At ~5 months post-hatch, fish were given unique identification by tagging with a passive integrated transponder (Avid Identification Systems Inc., Norco, CA) in the left-side dorsal musculature, and tagged fish were combined and reared in 800-L communal tanks supplied with partially-recirculated spring water (ambient temperature ~13 °C) until harvest at ~13 months post-hatch. Fish were fed a commercial fishmeal-based diet using automatic, programmable feeders (Arvotec, Huutokoski, Finland). The initial daily feeding rate in young fish was approximately 2.5% of body weight, and the daily feeding rate was gradually decreased to approximately 0.75% of body weight as fish grew. This feeding schedule is similar to that described previously^44^. Fish were starved for 5 days before harvest.

At fish sampling, WBW was measured in all fish belonging to 98 fish families then families were ranked descendingly based on their WBW. For muscle sampling, the 2^nd^ or 3^rd^ fish from each family was chosen to adjust the distribution of WBW around the median of the family. Selected fish were randomly assigned to one of five harvest groups (one fish/family/harvest group). The five harvest groups were sampled at ~13-month-old over 5 consecutive weeks (one group per each week, mean body weight = 985 g; SD = 239 g). In each of five consecutive weeks, approximately 100 fish (i.e., 1 fish per full-sib family per week) were anesthetized in approximately 100 mg/L of tricaine methane sulfonate (Tricaine-S, Western Chemical, Ferndale, WA) weighed, slaughtered, and eviscerated. A muscle sample was excised from the left dorsal musculature approximately midway between the head and dorsal fin and frozen in liquid nitrogen. Head-on gutted carcasses were packed in ice, transported to the West Virginia University Muscle Foods Processing Laboratory (Morgantown, WV), and stored overnight. The next day, carcasses were manually processed into trimmed, skinless fillets by a trained faculty member and weighed.

Muscle yield and fillet quality analyses were performed as previously described^45^. In brief, muscle yield was assessed as a percent of muscle weight relative to WBW. A muscle section (40 × 80 mm) was separated from the dorsal musculature for texture analysis. The Soxhlet solvent extractor with petroleum ether was used to analyze crude fat. Fillet texture was assessed using a five-blade, Allo-Kramer shear cell connected with a Texture Analyzer (Model TA-HDi®; Texture Technologies Corp., Scarsdale, NY), provided with a 50 kg load cell and at a crosshead speed of 127 mm/min. Texture Expert Exceed software (version 2.60; Stable Micro Systems Ltd., Surrey, U.K.) was used to record and analyze force-deformation graphs. Peak shear force (g/g sample) was recorded then families were ranked in a descending order. Fresh fillet surface color was measured with a Chroma Meter (Minolta, Model CR-300; Minolta Camera Co., Osaka, Japan), calibrated using a standard white plate No. 21333180 (CIE Y 93.1; x 0.3161; y 0.3326). L* (lightness), a* (redness), and b* (yellowness) values were recorded at three locations above the lateral line along the long axis of the right fillet, and these values were used to calculate a fillet whiteness index according to the following equation; Whiteness = 100 − [(100 − L)2 + a2 + b2]1/2^46^. The left-side fillet was frozen for subsequent proximate analysis, and a 4 × 8 cm fillet section was cut from the right side for subsequent cooked texture analysis. Details of the proximate and cooked texture analyses were previously described^47^.

### cDNA library construction and sequencing

RNA sequencing was done as we previously described^43^. Briefly, 98 fish families were ranked in a descending order according to the collected phenotypic data for each trait as described above. An average of eight different families (~5 fish each) showing opposite phenotypes for each of the five phenotypes were selected (4 high-ranked families versus 4 low-ranked families for each trait). Each family represents a full-sib family from the growth-selected line. Fillet tissue was collected from each fish and flash frozen in liquid nitrogen, and these tissues were then stored at −80 °C until total RNA isolation. Total RNA was isolated from each sample using TRIzol™ (Invitrogen, Carlsbad, CA). Quantity of total RNA was assessed by Qubit then the quality and integrity were checked by gel electrophoresis using Bioanalyzer 2100 (Aglient, CA). Total RNA from 5 samples of each family was used for RNA sequencing. Equal masses of total RNA from samples of each family were pooled and used for RNA sequencing. cDNA libraries were prepared and sequenced on Illumina HiSeq (single-end, 100 bp read length) using standard multiplexing protocols. Because some fish families were common between the traits, the total number of selected families for RNA-Seq was 22 families. Briefly, first-strand was synthesized with a random hexamer and SuperScript II (Life Technologies). Double stranded DNA was blunt-ended, 3′-end A-tailed, and ligated to indexed adaptors. The adaptor-ligated double-stranded cDNA was PCR-amplified for 10 cycles with the Kapa HiFi polymerase (Kapa Biosystems, Woburn, MA). The final libraries were Qubit-quantitated (Life Technologies, Grand Island, NY), and an Agilent bioanalyzer DNA7500 DNA chip (Agilent Technologies, Wilmington, DE) was used to determine the average size. Indexed libraries were pooled in equimolar concentration before sequencing using TruSeq RNA Sample Prep Kit v2 (Illumina, San Diego, CA).

### RNA-Seq expression analyses

Sequencing reads were trimmed using Trimmomatic to remove the adaptors sequences followed by FastQC quality control checks. Trimmed reads used for downstream analyses had quality score of Q30 or higher. For gene expression analysis, three references were combined for mapping the reads. Combined reference consisted of mRNAs identified in the rainbow trout genome reference^48^, and the newly annotated mRNA and lncRNA transcriptome references^49,50^. Albeit, the lncRNA reference needed to be improved to determine the orientation of lncRNA relative to the overlapping protein-coding genes. For this purpose, strand-specific RNA-Seq libraries from muscle (submitted to NCBI) and gill (GenBank Acc#SRP035242) were used to improve the lncRNA reference assembly basically as we previously described^49^. In brief, reads from muscle and gill tissues were mapped to the reference genome^48^ using TopHat. Cufflink was used to assemble the mapped reads into transcripts. Transcripts longer than 200 nt, and without coding potential and similarity to other noncoding RNA classes were considered as putative lncRNAs. Any single-exon lncRNA, adjacent to a protein-coding gene within 500 nt and in sense direction, was removed. All 25,516 newly identified, non-redundant lncRNA transcripts were merged with the old lncRNA reference yielding a total of ~51 k lncRNA transcripts (available at https://www.animalgenome.org/repository/pub/MTSU2017.1228/).

For quantification of expression of protein-coding genes and lncRNAs, sequencing reads from selected families were mapped to the reference using CLC genomics workbench (https://www.qiagenbioinformatics.com/). The CLC built-in, RNA-Seq analysis tool was used to generate expression tracks for transcripts. Statistical analysis using edgeR^51^ was performed on the expression values (Transcripts Per Kilobase Million; TPM) produced from RNA-Seq analyses to identify the DE genes. Genes with FDR < 0.05 and fold change value ≥ 2 or ≤−2 were considered as significant DE genes. The sequencing data is being submitted to the NCBI SRA database.

### Validation of DE genes by qPCR

To verify results obtained from RNA-Seq analyses, twelve DE protein-coding genes and five DE lncRNAs were chosen for validation by qPCR. Also, qPCR was used to validate association of eight transcripts with the reported phenotypes across 90 randomly selected samples. Primer3^52^ was used to design primers listed in Supplementary Table S1. To get rid of genomic DNA, RNA was treated with Optimize™ DNAase I (Fisher Bio Reagents, Hudson, NH) according to the manufacturer’s protocol. Reverse transcription reaction was performed to synthesize the first strand cDNAs via a Verso cDNA Synthesis Kit (Thermo Scientific, Hudson, NH) according to the manufacturer guidelines. qPCR was carried out by CFX96™ Real Time System (Bio Rad, Hercules, CA). Each qPCR reaction contained 2.5 μL template (100 ng/μL), 1 μL (10 μM working solution) forward and reverse primers, 5 μL SYBR Green master mix (Bio-Rad, Hercules, CA 94547), and 1.5 μL nuclease free water. A negative control reaction, without template, was performed for each primer to make sure that RNAs were free of genomic DNA. Sample analyses were replicated 3 times. *β*- actin gene was used as a control for normalization of expression. Only primers showing efficiency between 90 and 110% were used for qPCR. The PCR conditions for all reactions were 95 °C for 30 sec followed by 40 cycles. Each cycle started with 95 °C for 15 sec, followed by the appropriate annealing temperature for each primer for 30 sec, and completed at 60 °C for 30 sec. The delta delta Ct (ΔΔCt) method^28,53^ was used to quantify gene expression using qPCR data.

### Gene clustering and physical genomic localization

Expression values, TPM, of lncRNAs and protein-coding genes were used to generate gene clusters. Briefly, a scaling method in CLC genomics workbench was used to normalize the expression values of all transcripts. Normalized expression values of all transcripts in 22 trout families were uploaded to the Multi-Experiment Viewer (MeV) program^54–56^ to cluster protein-coding genes and lncRNAs at a minimum correlation threshold (R) of 0.85. In-house Perl scripts were used to classify lncRNAs according to relative location to their neighboring protein-coding genes on the rainbow trout genome^48,49^.

### Functional annotation and gene enrichment analysis

For functional annotation, Gene Ontology (GO) analysis of DE protein-coding genes was performed by Blast2GO^57,58^ and basic local alignment search against the KEGG database through KAAS-KEGG server Ver. 1.67x^59^, as we previously described^60^. Additionally, the Database for Annotation, Visualization and Integrated Discovery (DAVID) v6.8^61,62^ was used to perform gene enrichment analysis (Fisher Exact test p-value < 0.05) for protein-coding genes that are neighboring to and/or co-expressed with DE lncRNAs. The functional annotation chart of co-expressed genes was uploaded to EnrichmentMap plugin^63^ within the Cytoscape^64^ for visualization. The EnrichmentMap organizes the gene-sets (nodes), including pathways and Gene Ontology (GO) terms, into a network “enrichment map”. Overlapping gene-sets were clustered together with a FDR cutoff < 0.05 and overlap coefficient cutoff set at 0.5.

### Computational prediction of miRNA and lncRNA targets

For consensus miRNA target prediction, the DE lncRNAs and 3′ UTR of their co-expressed protein-coding genes were uploaded to the small RNA analysis server (sRNAtoolbox)^65^. The server has a pipeline for consensus animal miRNA target prediction. The pipeline uses three prediction tools for this purpose; miRanda, PITA, and TargetSpy. In addition to these three tools, “RNA22 version 2.0” was independently used as a fourth prediction tool to generate more reliable results. We considered the miRNA target when it had been predicted by at least three tools. The minimum free energy threshold of the microRNA: target hybridization was set at −13 Kcal/mol for all the tools. For lncRNA targets, DE lncRNAs and their co-expressed protein-coding genes were provided to a locally installed LncTar program^66^. The normalized deltaG (ndG) cutoff was set at −0.10.

### Identification of putative transcription factor binding sites (TFBS)

Promoter regions of DE lncRNA and their co-expressed protein-coding genes were scanned for putative TFBS of 26 transcription factors that are known to be involved in skeletal muscle development. These transcription factors are myogenin, MyoD, NF-AT1, c-Fos, c-Jun, JunB, FOXO4, CREB, Elk-1, E47, MAZ, MEF-2C, GATA-2, NFI/CTF, NF-Y, VDR, Smad3, Smad4, PEA3, SRF, Sp1, Sp3, YY1, p53, GR, and AR. An in-house Perl script was applied to retrieve 500 upstream nucleotide sequences of DE lncRNA and their co-expressed protein-coding genes. Extracted promoter sequences were uploaded to the ALGGEN server to find TFBS using PROMO software^67,68^. Parameters used were a maximum dissimilarity rate of 5% and a RE query (expectation of finding each of the matching motifs in a random sequence) <0.05.

## Results and Discussion

### Phenotypic Variation in population

**Figure 1:**
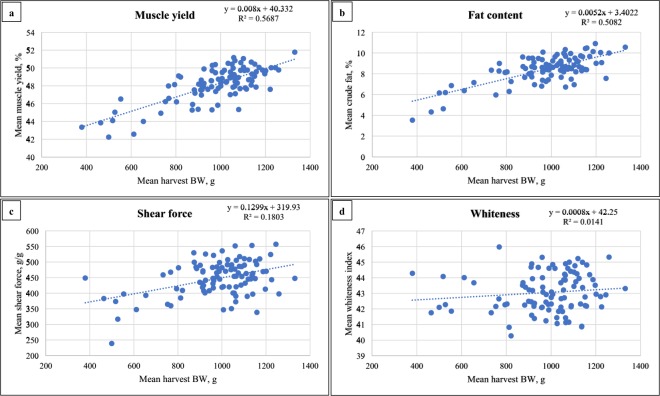
Effect of WBW as a variable on muscle yield and other quality traits (fat content, shear force and whiteness index) variations. WBW showed moderate regression coefficient (R^2^) values of 0.56 and 0.50 with muscle yield and fat content, respectively. Fillet whiteness and shear force had low coefficient values of 0.18 and 0.01 with WBW, respectively.

Genes involved in controlling fish muscle growth and quality were explored by characterizing global gene expression of mRNA and lncRNA in rainbow trout families revealing variations in WBW, muscle yield, and fillet quality traits (fat content, shear force, and whiteness index). In this study, variations in muscle yield and quality traits were characterized in fish from a growth-selected line at NCCCWA breeding program (after three generations of selection). To account for effects of WBW as a variable that may contribute to our interpretation of muscle yield and quality data, we performed a multivariable regression analysis using a mixed model. The model included random family effect, fixed sex effect and harvest dates and WBW was included as a linear covariate. Muscle yield and fat content showed moderate regression coefficient (R^2^) values of 0.56 and 0.50 with WBW, respectively (Fig. 1a,b). On the other hand, shear force and fillet whiteness had low coefficient values of 0.18 and 0.01, respectively (Fig. 1c,d). Our previous studies indicated potential genetic association between fast growth and increased muscle yield, paler fillets, and firmer texture^69^. Fillet paleness increases with increasing fat content. Moreover, we previously estimated moderate to high heritability for muscle yield, muscle weight, carcass weight, fat percentage, shear force, and fillet color; these estimated heritabilities imply existence of substantial additive genetic variation for growth and carcass traits in the population^69,70^.

**Figure 2:**
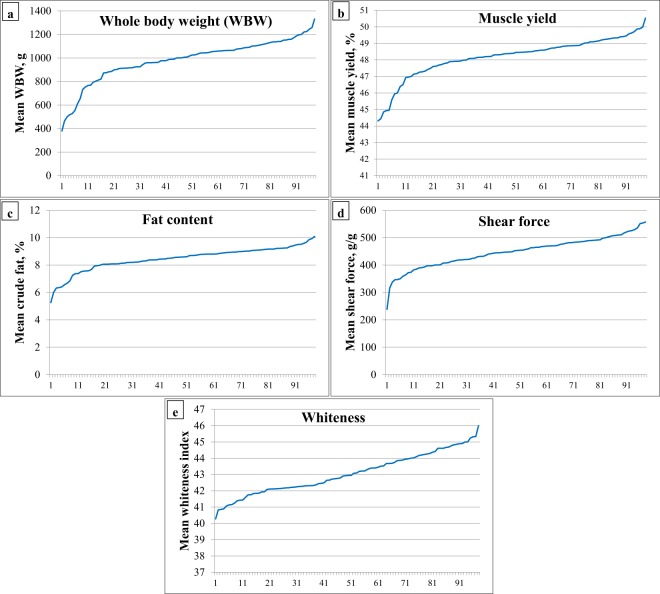
Phenotypic variation in growth and muscle quality traits in 98 families (~5 fish each).

To ensure that the gene expression association detected in muscle yield and fat content was not confounded by WBW, phenotypic values of the muscle yield and fat content were corrected for WBW, and the corrected values were used to select families with contrasting variations in muscle yield (49.5% of BW ± 1.1 vs. 44.8% of BW ± 1.7) and fat content (9.6% ± 0.9 vs. 6.3% ± 1.0). Phenotypic variation in growth and muscle quality traits in 98 families (~5 fish each) are shown in Fig. 2. Mean, standard deviation and phenotypic coefficient of variation for families showing divergent phenotypes (Average 4 high- vs. 4 low-ranked families) in each trait are listed in Table S2. Divergent phenotypic differences were statistically significant (P < 0.01).

### Analysis of RNA-Seq data and identification of DE protein-coding and lncRNA genes

**Figure 3:**
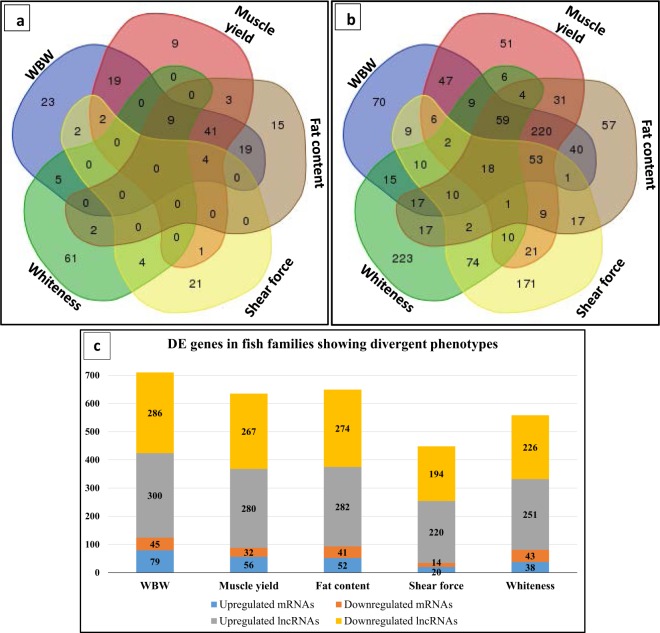
Venn diagram showing unique DE protein-coding genes (**a**) and DE lncRNAs (**b**) for each trait in addition to common genes between different traits. Number of DE genes for each trait is shown in (**c**) (FDR < 0.05 and fold change ≥ 2 or ≤−2).

**Figure 4:**
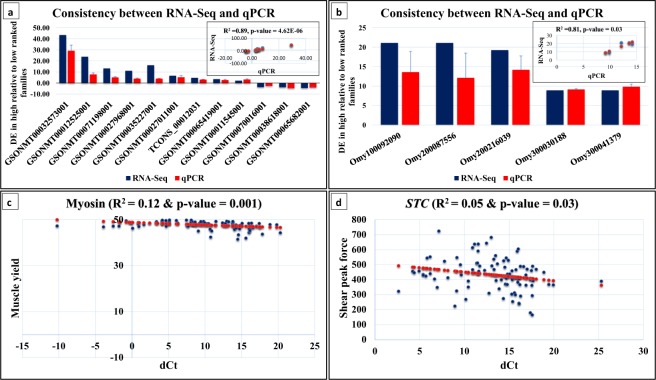
Consistency between RNA-Seq and qPCR measurements of DE protein-coding genes (**a**) and DE lncRNAs (**b**) (R^2^ = 0.89 & 0.81, respectively). Correlations between expression pattern of *MYSS* (**c**) and *STC* (**d**) with muscle yield and shear force, respectively.

RNA sequencing of 22 fish families yielded a total of 259,634,620 raw sequence reads (average of 11,801,573 reads/family) with ~9.3 depth of coverage. Sequencing reads were trimmed/filtered to generate 250,303,394 high quality reads. A total of 219,459,206 (87.68%) trimmed reads were mapped to the reference with ~7.3 depth of coverage. Quality and mapping statistics of sequencing reads are provided in Table S3. To identify DE genes, high- and low-ranked families for each trait were subjected to unpaired-comparisons using the CLC Genomics workbench. A total of 240 and 1,280 non-redundant protein-coding and lncRNA genes, respectively, were DE in all studied traits with FDR < 0.05 and a minimum fold change value ≥ 2 or ≤−2. Out of 1,280 DE lncRNAs, 1,061 novel lncRNA transcripts were identified in this study (Table S4). In agreement with a previous work, a higher variability in the expression of lncRNA compared with the protein-coding genes was observed^28,71^. Regression analysis showed a high Pearson correlation coefficient (R^2^ = 0.89) between number of DE protein-coding genes and DE lncRNAs in fish families showing contrasting phenotypes. Figure 3(a,b) shows Venn diagrams of the DE genes; WBW, muscle yield and fat content exhibited a large number of common, DE protein-coding genes (n = 41) and DE lncRNAs (n = 220). These results are consistent with previously reported pleiotropic and epistatic effects of genomic loci on fat and muscle weight controlling WBW as a composite trait^72^. Whereas, whiteness and shear force exhibited a large number of unshared DE protein-coding and lncRNA genes. Shear force and whiteness displayed fewer DE protein-coding genes and DE lncRNAs compared to the other traits. Detailed information about DE genes are shown in Fig. (3c) and Supplementary Tables 4 and 5. There was a significant correlation between transcript fold-change values determined by RNA-Seq and qPCR (R^2^ = 0.89 for protein-coding genes and 0.81 for lncRNAs; Fig. 4a,b, respectively).

### Annotation of DE protein-coding genes in families showing divergent phenotypes

GO analysis showed that DE protein-coding genes were highly represented in macromolecule metabolic process (n = 119) (Table S6). Enrichment analysis of those genes (Fisher Exact test p-value < 0.05) is provided in Table (S7). In addition, several transcripts were involved in growth-associated mechanisms such as protein metabolism (n = 90), muscle structure development (n = 52), lipid metabolism (n = 34), and oxidation-reduction processes (n = 22) (Table S6). Similar GO annotations were previously observed in genes associated with muscle growth and quality traits in rainbow trout^14,73–75^.

In the current study, several genes (n = 46) that are involved in either proteolytic processes or cell growth were significantly DE in fish families showing contrasting phenotypes (Table S5). Out of them, 17 transcripts had functions related to protein ubiquitination, autophagy, proteolysis, or lysosome activity (Table S5). For example, we identified five transcripts encoding F-box only 32 (Atrogin-1), an E3 ubiquitin ligase, were upregulated in families showing low WBW (Table 1). High levels of Atrogin-1 stimulate protein degradation and suppress protein synthesis^76^. All Atrogin-1 transcripts showed a similar pattern of expression. By aligning the five transcripts to the trout genome, it turned out that they are partial sequences mapped to a single genomic locus indicating transcript misassembly. On the other hand, 14 out of 46 transcripts were categorized as transcription regulators (Table S5); of them, ankyrin repeat domain-containing protein 1 (*ANKRD1*) and gremlin-1 showed the highest fold changes (Table 1). *ANKRD1* was downregulated whereas gremlin-1 was upregulated in fish families with high WBW and fat content. Previously, gremlin-1 has been reported as a regulator of proliferation and differentiation of myogenic progenitors in skeletal muscle^77^. The list of this category also includes connective tissue growth factor (CTGF) which enhances proliferation/differentiation, and proteasome-associated ECM29 homolog that enhances proteolysis; both showed DE in fish families exhibiting high whiteness index (Table 1). More details about other DE transcripts involved in cell growth/proliferation were provided in Supplementary Table (S5).

**Table 1:** A subset of differentially expressed protein-coding genes (FDR < 0.05) in fish families showing contrasting phenotypes.

Feature ID	Annotation	Fold change/trait (high/low)	FDR/trait	Trait
**Genes involved in protein ubiquitination and growth**				
TCONS_00058870	F-box only protein 32	−3.61|−3.16|−3.39	2.5E-02|4.0E-02|4.3E-02	WBW|Mus%|Fat%
TCONS_00058871	F-box only protein 32	−3.14	4.9E-02	WBW
TCONS_00098636	F-box only protein 32	−2.71	4.6E-02	WBW
GSONMT00016768001	F-box only protein 32	−2.36	3.20E-09	WBW
GSONMT00031929001	F-box only protein 32	−2.19	2.1E-06	WBW
GSONMT00065477001	Ankyrin repeat domain-containing protein 1	−6.32|−4.99|−6.63	2.6E-06|2.4E-05|3.2E-06	WBW|Mus%|Fat%
GSONMT00041801001	Gremlin-1	4.63|4.32	3.9E-02|3.9E-02	WBW|Fat%
GSONMT00011893001	Connective tissue growth factor	−9.19	3.1E-06	Whiteness
TCONS_00152966	Proteasome-associated ECM29 homolog	6.02	2.1E-02	Whiteness
**Genes involved in fat metabolism**				
GSONMT00070016001	Caveolin-3	−3.29|−3.25|−3.73	2.00E-04|2.00E-04|1.04E-04	WBW|Mus%|Fat%
GSONMT00000701001	5-AMP-activated protein kinase subunit γ-2	−4.14|−3.68|−3.86	3.5E-02|3.5E-02|3.5E-02	WBW|Mus%|Fat%
GSONMT00038618001	Endophilin-b1 isoform x1	−3.86	6.4E-04	Fat%
GSONMT00017014001	Lymphocyte g0 g1 switch protein 2	4.47|3.66|3.84	3.8E-06|4.5E-04|2.2E-04	WBW|Mus%|Fat%
GSONMT00019603001	Hormone-sensitive lipase isoform x2	2.6	2.70E-02	Fat%
GSONMT00076211001	Perilipin-1	2.62|2.36|2.64	5.0E-11|3.8E-08|4.5E-11	WBW|Mus%|Fat%
GSONMT00054194001	Lipoprotein lipase	2.62|2.19|2.46	2.2E-06|1.0E-03|3.9E-05	WBW|Mus%|Fat%
GSONMT00079455001	Apolipoprotein A-IV	3.51|3.29|3.27|2.95	2.4E-12|1.2E-10|1.3E-10|2.9E-10	WBW|Mus%|Fat%|Whiteness
GSONMT00000920001	Fatty acid-binding heart	2.45|2.23|2.25	1.9E-39|8.9E-31|3.2E-31	WBW|Mus%|Fat%
GSONMT00080511001	Adipocyte plasma membrane-associated protein	2.69|2.23|2.58|2.03	9.2E-09|5.9E-05|1.0E-07|2.4E-05	WBW|Mus%|Fat%|Whiteness
GSONMT00075321001	Diacylglycerol o-acyltransferase 2	2.85|2.38|2.73	2.3E-03|4.3E-02|5.3E-03	WBW|Mus%|Fat%
**Structural genes**				
GSONMT00032573001	Myosin heavy chain	43.33|43.40	3.1E-02|3.1E-02	WBW|Mus%
GSONMT00065900001	Troponin fast skeletal muscle	99.04|72|49.87|2.99	2.6E-10|4.0E-07|1.3E-04|2.8E-02	WBW|Mus%|Fat%|Shear force
TCONS_00057247	Troponin fast skeletal muscle	12.82|13.64	3.0E-07|5.9E-08	WBW|Mus%
GSONMT00023675001	Troponin fast skeletal muscle	−2.38|−2.17|−2.1	6.3E-101|3.8E-85|2.4E-82	WBW|Mus%|Fat%
GSONMT00065895001	Troponin fast skeletal muscle	−2.75|−2.71|−2.27|−2.25	0.0E + 00|0.0E + 00|2.2E-236|5.9E-192	WBW|Mus%|Fat%|Shear force
**Genes involved in calcium metabolism**				
GSONMT00012525001	Stanniocalcin	23.75	1.40E-06	Shear force
TCONS_00012355	Stanniocalcin	12.82	2.2E-05	Shear force
GSONMT00027968001	Stanniocalcin	11.08	1.84E-08	Shear force
GSONMT00063580001	Parvalbumin	−4.2|−4.89|−3.97|−6.02	1.8E-104|5.1E-119|3.4E-99|3.1E-136	WBW|Mus%|Fat%|Shear force
**Genes involved in oxidative stress**				
GSONMT00020998001	Thioredoxin	3.25|2.66|3.05	2.0E-15|2.7E-09|3.4E-13	WBW|Mus%|Fat%
GSONMT00070684001	Glutathione peroxidase 1	2.62|2.62|2.81	3.7E-24|5.5E-24|1.4E-28	WBW|Mus%|Fat%

Fold change, in families of high phenotype relative to th ose of low phenotypes, ≥ 2 or ≤2 are shown.

Many DE genes (n = 30) were associated with fat metabolism. Of them, 17 transcripts were DE in families with variations in fat content (Table S5). Caveolin-3, 5-AMP-activated protein kinase subunit gamma-2 (*AAKG2*), and endophilin-b1 were downregulated, and seventeen other transcripts, including lymphocyte g0 g1 switch protein 2 (*G0S2*), hormone-sensitive lipase, perilipin-1 (*PLIN1*), lipoprotein lipase (*LIPL*), apolipoprotein A-IV, fatty acid-binding heart (*FABPH*), adipocyte plasma membrane-associated protein (*APMAP*), and diacylglycerol o-acyltransferase 2 were upregulated in families exhibiting high fat content (Table 1). Based on our KEGG pathway analysis, *CAV3* was involved in the focal adhesion pathway. Cell junction-related pathways that include focal adhesion and preserve tissue integrity were enriched along with lipid metabolism pathways in fast- and slow-growing chicken breeds suggesting a role in intramuscular fat deposition^78^. Previous studies showed association of *APMAP*^79,80^, diacylglycerol O-acyltransferase 1 (*DGAT1*)^81^ and *PLIN1*^82–88^ with adiposity and carcass traits. Anti- *APMAP* antibodies was used to decrease the backfat and increase the lean meat percentage in pigs and other animals^79,80^. It is worth mentioning that seven of these fat metabolism associated genes were DE in fish families of contrasting whiteness index suggesting a role of fat in determining fillet color (Table S5).

Several structural genes (n = 50) showed differential expression in families of divergent phenotypes. For example, 14 transcripts encoding myosin heavy, fast and slow chains in addition to two transcripts encoding myosin light chain-3 and -4 exhibited differential expression in fish families of contrasting phenotypes (Table S5). To investigate whether transcripts of myosin heavy chain represent different isoforms generated as a result of the alternative splicing events, we mapped them to the trout genome. The transcripts were mapped to of six unique genomic loci, some represent partial/incomplete myosin sequences as a result of transcriptome misassembly. To emphasize the correlation between myosin heavy chain (*MYSS*) (GSONMT00032573001) and muscle yield, we quantified the abundance of *MYSS* transcripts in 90 fish using qPCR; these fish were randomly chosen from the population sample evaluated in this study. *MYSS* had an R^2^ value of 0.12 (p-value = 0.001), suggesting a significant role of *MYSS* in explaining variation in muscle yield (Fig. 4c). Other transcripts necessary for the muscle mass and muscle contraction were also included in the DE list. For example, seven transcripts encoding three regulatory subunits of troponin complex (Troponin I, C, and T) were DE. Two transcripts encoding troponin I, fast (*TNNI2*) were significantly upregulated in families showing high WBW and muscle yield whereas two other *TNNI2* transcripts were downregulated in these traits (Table 1). Troponin isoforms have an impact on the muscle fiber characteristics and could be used to improve quality traits in selection programs^89^. To test if troponin could be used as a potential biomarker for muscle quality traits in rainbow trout, we quantified the highly upregulated transcript of *TNNI2* (GSONMT00065900001) across 90 random fish samples. *TNNI2* (GSONMT00065900001) showed significant association with WBW (R^2^ = 0.10; p-value = 0.008), muscle yield (R^2^ = 0.06; p-value = 0.02), and fat content (R^2^ = 0.15; p-value = 0.0003). Skeletal muscle *TNNI* was suggested to be used as a biomarker to identify fat adulteration^90^. More details about other troponin subunits were provided in Supplementary Table (S5). Further, 27 other structural transcripts were DE; 10 of them were upregulated in high WBW and muscle yield fish families. These transcripts included type II keratin E3, nebulin, PDZ and LIM domain 5, tropomyosin alpha-3 chain, slow myotomal muscle tropomyosin, and type I cytoskeletal-13 (Table S5).

Interestingly, three transcripts of stanniocalcin (*STC*; GSONMT00027968001, GSONMT00012525001 & TCONS_00012355) were highly overexpressed in families of high shear force (Table 1). The three transcripts were mapped to a single genomic locus in the trout genome. *STC* is the main regulatory hormone of Ca^+2^ homeostasis in fish^91^. Calcium is essential in regulating post-mortem muscle tenderization, at least partially, through activating the Ca^2+^-dependent cysteine proteases (Calpains)^92^. Similar to *MYSS* (GSONMT00032573001) and *TNNI2* (GSONMT00065900001), qPCR regression analysis showed that *STC* had R^2^ value of 0.05 (p-value = 0.03) indicating a potential role of *STC* in explaining shear force variation (Fig. 4d). However, Ca^+2^ analyses showed insignificant difference (p-value = 0.09) in muscle tissue of fish from the 4^th^ generation (an average of 60.83 umol/g dry versus 48.71 umol/g dry in families of high and low shear force, respectively, data not shown). Here we reported 19 other DE protein-coding genes that bind to Ca^+2^ ions such as parvalbumin (*PV*) (Table S5). Opposite to *STC*, *PV* (GSONMT00063580001) was the highly downregulated transcript in fish of high shear force and muscle yield (Table 1). *PV* was previously suggested as a biomarker for muscle mass and tenderness^93,94^.

Thioredoxin (*THIO*) and glutathione peroxidase-1 (*GPXl*) were upregulated in families of high WBW, muscle yield and fat content (Table 1). These results agree with previous studies reported that *THIO* has antioxidant and antiapoptotic properties and induces autocrine cell growth^95^ while *GPXl* prevents fat oxidation that deteriorates fillet flavor and color^96^. We also identified other DE transcripts involved in oxidation-reduction reactions and having oxidoreductase activity. These transcripts are retinol dehydrogenase 11 (*RDH11*), dimethylaniline monooxygenase (*FMO5*), cytochrome c oxidase subunit VIb isoform 1 (*CX6B1*), very-long-chain enoyl-CoA reductase, and NADH-cytochrome b5 reductase 3 (*NB5R3*) (Table S5).

### Correlation between DE lncRNAs and protein-coding genes

Some lncRNA annotations are available only for human and other model species. LncRNAs are poorly conserved among species^97^ and this characteristic makes it hard to directly annotate lncRNAs, and consequently, anticipate their impact on the muscle growth and quality phenotypes. To tackle this difficulty, we studied the correlation between the DE lncRNAs and protein-coding genes according to their physical locations in the genome and expression correlation. According to the physical location, out of 1,280 DE lncRNAs, there were 368 genic and 912 intergenic lncRNAs. The genic lncRNAs were further subdivided as exonic (n = 112) or intronic (n = 256). More information about this classification is provided in Table (S8). Based on these criteria, DE lncRNAs and mRNAs were classified into the following two categories.

#### Correlated and overlapped DE lncRNAs and protein-coding genes

**Figure 5:**
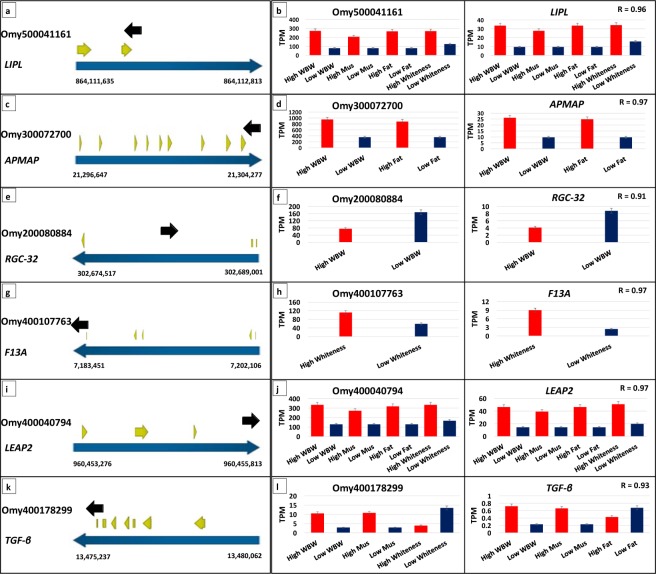
(**a**–**l**) Orientation of lncRNAs relative to the overlapping protein-coding loci (on the left) and comparison of the expression patterns (TPM) of DE lncRNAs with the overlapping DE protein-coding genes across families showing significant phenotypic variations (on the right).

Many lncRNAs act in *cis* configuration to regulate expression of their adjacent genes^98,99^. To identify the potential *cis*-acting regulatory lncRNAs in this study, we first identified the lncRNAs that were overlapping with protein-coding genes. There were 368 genic DE lncRNAs either fully (n = 13) or partially overlapped (n = 355) with protein-coding genes in sense or antisense orientation (Table S8). Second, to identify the probable relationships between the DE lncRNAs and their overlapping protein-coding genes, we compared their expression pattern across the 22 different families considered in this study. Normalized expression values (TPM) were used to generate gene clusters between DE lncRNA and protein-coding genes with a correlation coefficient value of R ≥ 0.85 (Table S9). Six DE lncRNAs were correlated in expression with six overlapping protein-coding genes (Table 2 and Fig. 5). Orientation of each lncRNA relative to its overlapping protein-coding locus was confirmed by strand-specific PCR. Association between two out of six pairs of DE lncRNA-mRNA and the phenotypes was validated by qPCR (Table 3).

**Table 2:** Differentially expressed lncRNAs (FDR < 0.05) showing correlation with overlapping and neighboring protein-coding genes existing within 50 kb.

LncRNA	Protein-coding gene	Annotation	Overlap	Direction	R	Trait
**Overlapping protein-coding genes & LncRNAs**						
Omy500041161	GSONMT00039165001	Lipoprotein lipase	Partial/Exonic	Antisense	Positive (0.96)	WBW/Mus%/Fat%/Whiteness
Omy300072700	GSONMT00080511001	Adipocyte plasma membrane-associated	Partial/Exonic	Antisense	Positive (0.97)	Fat%/WBW
Omy200080884	GSONMT00034829001	Response gene to complement 32 protein	Complete/Intronic	Antisense	Positive (0.91)	WBW
Omy400107763	GSONMT00082197001	Coagulation factor XIII A chain	Exonic	Sense	Positive (0.97)	Whiteness
Omy400178299	GSONMT00041090001	Transforming growth factor-beta	Exonic	Sense	Positive (0.93)	WBW/Mus%/Whiteness
Omy400040794	GSONMT00033306001	Liver-expressed antimicrobial peptide 2	Exonic	Sense	Positive (0.97)	WBW/Mus%/Fat%/Whiteness
**Correlating protein-coding genes & LncRNAs lie within 50 Kb**			**Distance (Kb)**			
Omy100092090	GSONMT00012525001	Stanniocalcin	1.456	Antisense/Intergenic	Positive (0.94)	Shear force
Omy400016750	GSONMT00061222001	DNA (cytosine-5)-methyltransferase 3 A	7.931	Antisense/Intergenic	Positive (0.88)	Whiteness
Omy500090683	GSONMT00056166001	Nuclear protein localization protein 4 homolog	1.162	Antisense/Intergenic	Positive (0.85)	WBW/Fat%
Omy200231682	GSONMT00079455001	Apolipoprotein A-IV	1.786	Unknown/Intergenic	Positive (0.90)	WBW/Mus%/Fat%/Whiteness
Omy500089619	GSONMT00002133001	s-adenosylmethionine synthase isoform type-1	0.670	Antisense/Intergenic	Positive (0.97)	WBW/Mus%/Fat%
Omy100162939	GSONMT00017975001	Myosin-6 isoform x1	18.744	Unknown/Intergenic	Positive (0.96)	Shear force
Omy100162939	GSONMT00017978001	Slow myosin heavy chain 1	38.362	Unknown/Intergenic	Positive (0.90)	Shear force
Omy500072095	GSONMT00067129001	Autophagy-related protein 9 A isoform x1	1.875	Antisense/Intergenic	Positive (0.85)	WBW/Fat%
Omy500086794	GSONMT00007952001	Ras-related protein Rab-1A	1.569	Antisense/Intergenic	Positive (0.96)	WBW
Omy500084299	GSONMT00066744001	Triadin- partial	19.644	Antisense/Intergenic	Positive (0.93)	WBW
Omy400001433	GSONMT00041695001	Calcium-binding and coiled-coil domain-containing protein 1	3.742	Sense/Intergenic	Positive (0.99)	WBW/Whiteness
Omy400042056	GSONMT00031929001	F-box only protein 32	1.260	Sense/Intergenic	Positive (0.88)	WBW

**Table 3:** Association between expression of two overlapping and co-expressed, DE lncRNA-mRNA pairs (Omy500041161-*LIPL* and Omy400178299-*TGF-β*) and muscle quality traits validated by qPCR across 90 randomly selected individual fish.

Transcript ID	WBW (R^2^, p-value)	Muscle% (R^2^, p-value)	Fat% (R^2^, p-value)	Shear force (R^2^, p-value)	Whiteness (R^2^, p-value)
Omy500041161	0.02 (0.24)	0.01 (0.43)	0.11 (0.01)*	0.0044 (0.59)	0.00067 (0.83)
GSONMT00039165001 (*LIPL*)	0.21 (0.000086)*	0.07 (0.03)*	0.24 (0.000019)*	0.01 (0.4)	0.02 (0.23)
Omy400178299	0.09 (0.01)*	0.04 (0.1)	0.01 (0.37)	0.03 (0.18)	0.01 (0.58)
GSONMT00041090001 (*TGF-β*)	0.08 (0.01)*	0.04 (0.08)	0.05 (0.06)	0.00021 (0.9)	0.01 (0.48)

* indicates a significant p-value < 0.05.

Interestingly, in five (out of six) lncRNA-mRNA pairs, lncRNA and its counterpart protein-coding gene were DE in association with specific traits (FDR < 0.05). In the first pair, lncRNA Omy500041161 was partially overlapped with the second exon of the *LIPL* gene (GSONMT00039165001) in an antisense orientation, and their expressions were positively correlated (R = 0.96); both transcripts were upregulated in families exhibiting high WBW, muscle yield, fat content, and whiteness (Fig. 5a,b). qPCR analysis revealed a significant association between the first pair (Omy500041161/*LIPL*) and the fat phenotype across 90 random fish samples (Table 3). LncRNA target prediction analysis showed that lncRNA Omy500041161 targets the overlapping *LIPL* gene with high-confidence cutoff values (free energy^1^ of −396.84, and normalized binding free energy [ndG] = −198.42). Furthermore, lncRNA Omy500041161 and its overlapping gene (*LIPL*) shared transcription factor binding sites for androgen receptor (*AR*) and vitamin D receptor (*VDR*). The *AR* and *VDR* contribute to skeletal muscle development^100^ and function^101^. In agreement with our work, *LIPL* was upregulated in fast- versus slow-growing chickens^102^. Hydrolysis of circulating triglycerides and very low-density lipoproteins by *LIPL* produce free fatty acids that could be stored as neutral lipids in adipose tissue or used as an energy source by skeletal muscle^103^. In the second pair, lncRNA Omy300072700 was partially overlapped with the last 3′ exon of the *APMAP* gene (GSONMT00080511001) in an antisense orientation and exhibited positively correlated expressions (R = 0.97). These two transcripts were upregulated in families with high WBW and fat content (Fig. 5c,d). Interestingly, the antisense lncRNA (Omy300072700) was predicted to compete with its co-expressed/overlapping target *APMAP* (GSONMT00080511001) in binding mir-26a and mir-4185 (Table S10). In the third pair, lncRNA Omy200080884 was completely overlapped in antisense direction with the first 3′ intron of the gene coding for response gene to complement 32 protein (*RGC-32;* GSONMT00034829001). The two transcripts showed positively correlated downregulation in families with high WBW (R = 0.91) (Fig. 5e,f). *RGC-32* is a downstream target of transforming growth factor-beta (*TGF-β*)^104^. In the fourth pair, lncRNA Omy400107763 was completely overlapped in sense orientation with the first 3′ exon and part of first 3′ intron of coagulation factor XIII A chain gene (GSONMT00082197001). Both transcripts showed strong positive expression correlation (R = 0.97). The transcripts were upregulated in families showing high whiteness index (Fig. 5g,h). In the fifth pair, lncRNA Omy400040794 was partially overlapped with the 3′ UTR of the gene coding for liver-expressed antimicrobial peptide 2 (*LEAP2*) (GSONMT00033306001) in sense direction with strong positive expression correlation (R = 0.97) (Fig. 5i). These transcripts were upregulated in high versus low families of WBW, muscle yield, fat content, and whiteness (Fig. 5j). It has also been reported that the use of antimicrobial compounds increases the shelf life and quality of the fillet^105^. Further studies are needed to explore the roles of these lncRNAs in regulating their protein-coding counterparts.

Also, DE lncRNAs correlated with overlapping non-DE protein-coding genes. DE lncRNA Omy400178299 was positively correlated (R = 0.93) with non-DE *TGF-β* (GSONMT00041090001) across the 22 families. Overlap was in a sense orientation of the *TGF-β* sixth and seventh exons (Fig. 5k,l). The microRNAs (mir-10b and mir-181d) were predicted to target Omy400178299 and its co-expressed/overlapping *TGF-β gene* (GSONMT00041090001) (Table S10). Elevated levels of *TGFβ* and its downstream mediators (Smad 2, 3 and 4) were correlated with high levels of miR-181d^106^ and mir-10b^107^ in cancer cells. Remarkably, qPCR analysis showed a significant association between Omy400178299/*TGF-β* pair and WBW phenotype across 90 individual fish selected randomly (~R2 = 0.09; Table 3). Also, DE lncRNA Omy400037611 was correlated with overlapping non-DE protein-coding gene (R = 0.85) that codes for GTPase IMAP family member 4 isoform (GSONMT00033945001).

#### Correlated and non-overlapping DE lncRNAs and protein-coding genes

DE lncRNAs within this category have been subdivided into two groups:

*Cis*-acting DE lncRNAs: As mentioned earlier, 912 out of 1,280 DE lncRNAs were categorized as intergenic, and they did not overlap with protein-coding genes. To identify lncRNAs with a potential *cis*-regulatory effect to non-overlapping neighboring genes, we searched for DE lncRNAs with protein-coding genes on both sides within a distance of 50 kb.

The 912 DE lncRNAs had 841 protein-coding genes within 50 kb. Gene enrichment analysis of the neighboring genes (Fisher Exact test p-value < 0.05) revealed that 111 genes (13.2%) were enriched in 10 KEGG pathways. These pathways include focal adhesion, insulin-signaling, ERbB signaling, phosphatidylinositol signaling, FoxO signaling, JAK-STAT signaling, and mTOR signaling. Further, epidermal growth factor domain and insulin-like growth factor binding protein were enriched (Table S11). These signaling pathways are involved in regulating skeletal muscle growth/mass^108,109^.

Interestingly, 11 DE lncRNAs were co-expressed with twelve neighboring protein-coding genes of a potential importance to muscle growth and quality. Transcripts encoding *STC* (GSONMT00012525001) and Omy100092090 were about 1.5 kb away from each other, and their expression was positively correlated (R = 0.94) (Table 2). LncRNA Omy100092090 and *STC* were DE in association with muscle shear force. Additionally, a transcript coding for DNA (cytosine-5)-methyltransferase 3 A (*DNMT3a*) (GSONMT00061222001), located within 7.9 kb from DE lncRNA (Omy400016750), exhibited positive expression correlation (R = 0.88). These two transcripts were upregulated in families with a high whiteness index (Table 2). Previous studies showed that the DNA methylation gene, *DNMT3a*, was highly expressed in skeletal muscle and significantly associated with quality traits^110–112^. A single transcript encoding *FBXO32* (Atrogin-1_ (GSONMT00031929001) and DE lncRNA Omy400042056, located within 1.3 kb, were positively correlated (R = 0.88; Table 2). *FBXO32* and Omy400042056 were downregulated in fish families of high WBW. This result agrees with a previous study in rainbow trout that indicated upregulation of *FBXO32* was associated with muscle atrophy^113^. Remaining and neighboring co-expressed DE lncRNAs and muscle relevant protein-coding genes are shown in Table 2.

**Figure 6:**
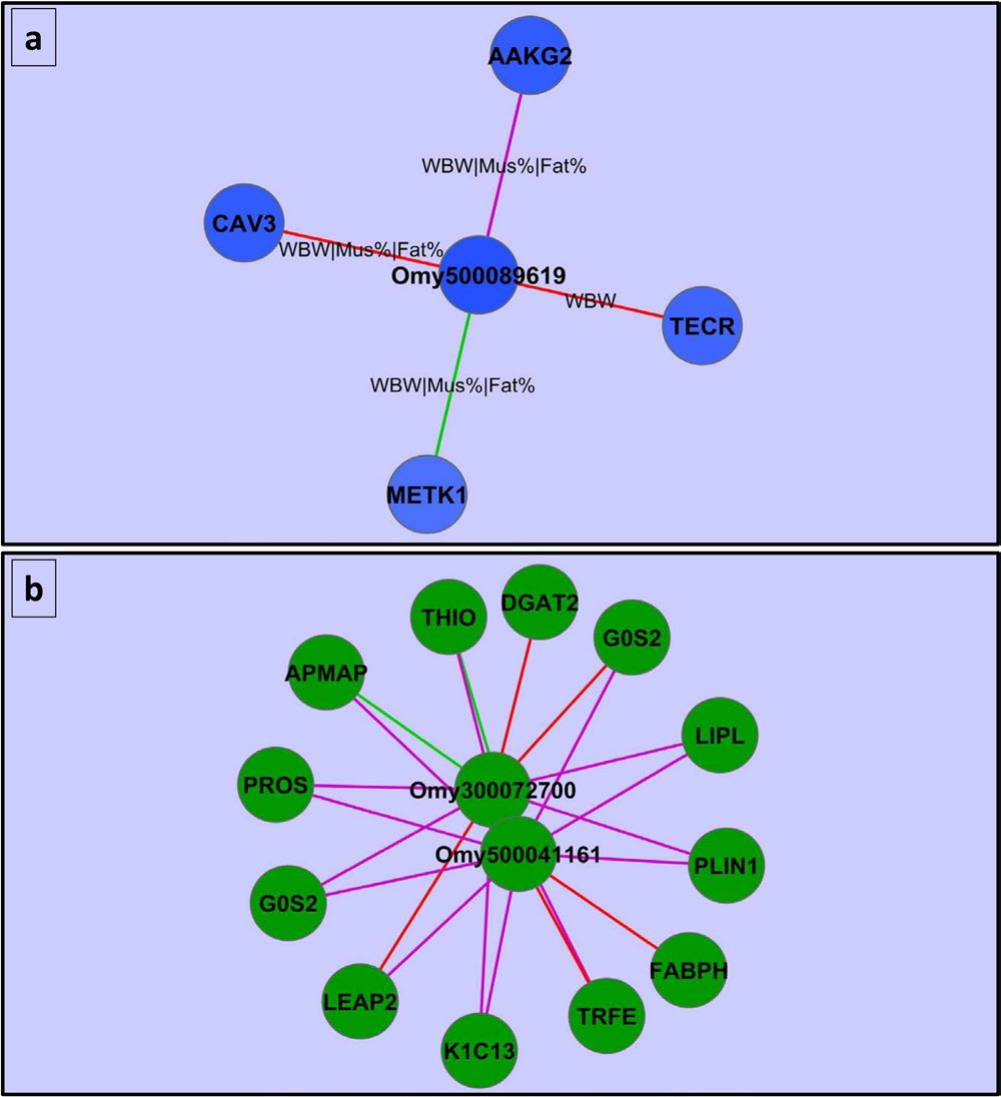
(**a**) Correlation between single DE lncRNA and co-expressed distant protein-coding genes in addition to their correlations with the phenotypes (WBW, muscle yield, and fat content). The color intensity of the nodes reflects the correlations between the genes and phenotypes. The color of the edges reflects the correlation between the protein-coding genes and DE lncRNA where red denotes 0.90 > R ≥ 0.85, purple color denotes 0.95 > R ≥ 0.90, and green color means R ≥ 0.95. (**b**) Correlation between two antisense DE lncRNAs and their co-expressed distant protein-coding genes that are significantly upregulated in fish families showing variations in WBW, muscle yield, and fat content and known to have an impact on the phenotypes. Edge colors reflect the strength of expression correlation between the DE lncRNAs and protein-coding genes.

*Trans*-acting DE lncRNAs: It has been shown that lncRNAs can work in both *cis* and *trans* configuration^114,115^ to regulate protein-coding genes located distantly on the same or a different chromosome. To determine their expression correlation, DE lncRNAs and protein-coding genes were clustered together according to their expression values across 22 families. Several clusters, with expression correlation (R) ≥ 0.85, have been identified between DE lncRNAs and all protein-coding genes, including DE protein-coding genes, which are distantly distributed in the trout genome (Table S9). These clusters include correlations between single lncRNAs and several different protein-coding genes as previously reported^28,116^. Cytoscape platform was used to visualize molecular interaction networks among the whole set of co-expressed genes and the phenotypic traits. We detected that, among all the 229 co-expressed lncRNAs, lncRNA (Omy500089619) exhibited the highest negative correlation with WBW, muscle yield, and fat content phenotypes across 22 families. qPCR analysis, across 90 individuals, revealed a significant association between Omy500089619 and the WBW, muscle yield, and fat content phenotypes (R^2^ = 0.09 (p-value = 0.02), 0.09 (p-value = 0.02), and 0.15 (p-value = 0.002), respectively). In addition, four protein-coding genes, co-expressed with Omy500089619, showed the highest negative correlations with the aforementioned three phenotypic traits (Fig. 6a). These protein-coding genes are *CAV3* (GSONMT00070016001), very-long-chain enoyl-CoA reductase (GSONMT00029837001; TECR), s-adenosylmethionine synthase isoform type-1 (GSONMT00002133001; METK1), and *AAKG2* (GSONMT00000701001). The *AAKG2* acts as a metabolic master switch that turns on fatty acid oxidation by acetyl-CoA carboxylase-2 phosphorylation and turns off fatty acid synthesis by acetyl-CoA carboxylase-1 phosphorylation^117^. The list also includes the lncRNAs Omy500041161 and Omy300072700 that were upregulated in families showing variations in WBW, muscle yield, and fat content. These DE lncRNAs were co-expressed across 22 families with distantly located and upregulated protein-coding genes that impact fillet quality; these upregulated protein-coding genes are *APMAP, G0S2, THIO, DGAT2, FABPH, PLIN1, LEAP2*, vitamin K-dependent protein S (*PROS*), serotransferrin (*TRFE*)*, and LIPL* (Fig. 6b).

### Gene enrichment analysis of the lncRNA co-expressed protein-coding genes

**Figure 7:**
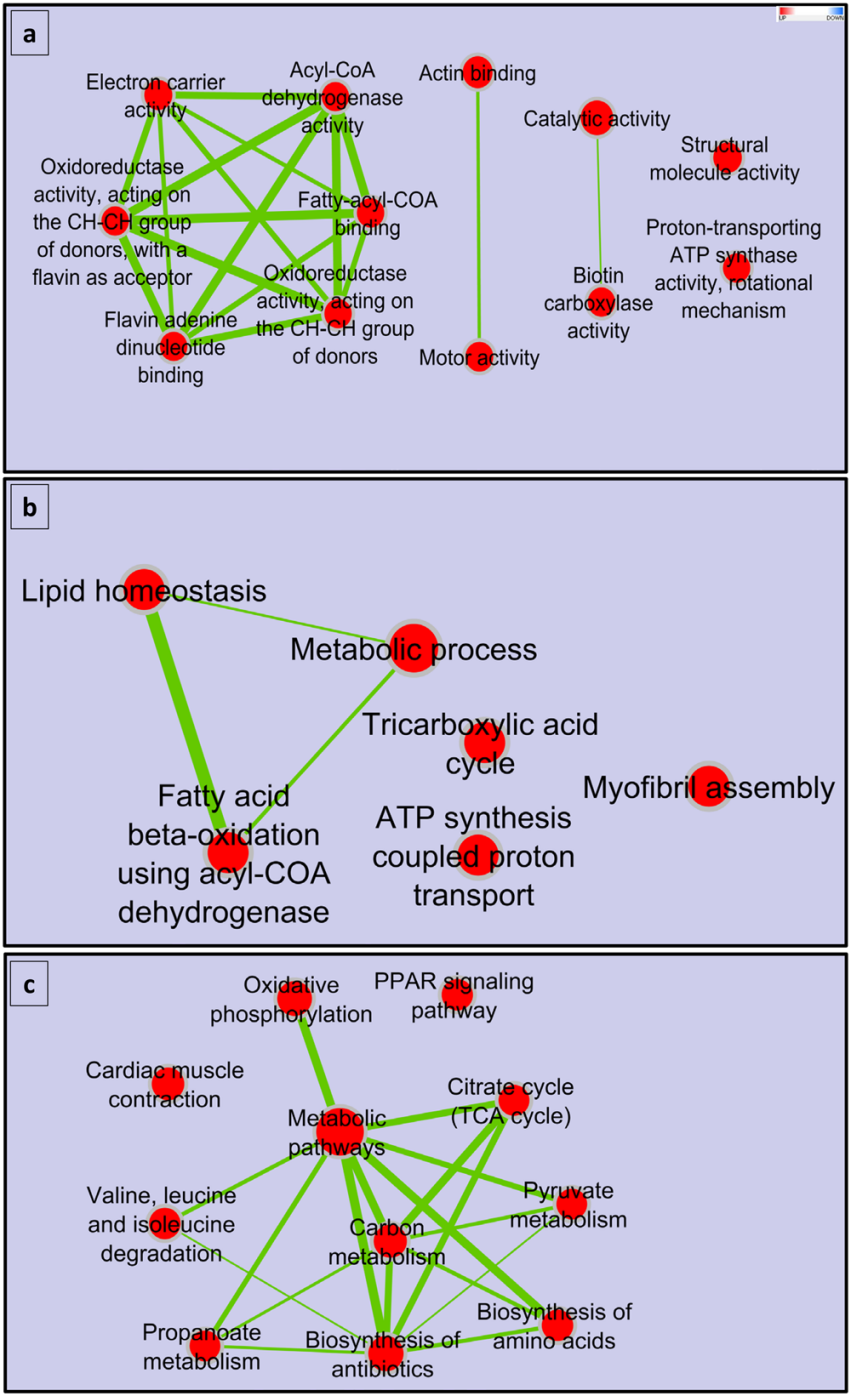
Gene enrichment analysis of protein-coding genes co-expressed with DE lncRNAs. Enriched gene-sets are represented as red nodes connected according to their GO/KEGG pathway relations. Color intensity of the node represents the fold enrichment while node size represents number of genes in the gene-set. Enriched terms belonging to metabolic pathways, energy and growth-related mechanisms were predominant.

Co-expression analysis across 22 families, with Pearson’s correlation coefficients of R ≥ 0.85, was performed to predict the probable targets of lncRNAs in *cis/trans*-regulatory relationships. A total of 3,392 positive correlations (edges) were successfully detected. Gene enrichment analysis using DAVID^61,62^ was performed to determine the probable functions of co-expressed genes and infer the mechanism of gene regulation by lncRNAs (Fig. 7). Functional annotation revealed 279 genes with enriched GO terms under the biological process. These genes are involved in cell adhesion, the ubiquitin-dependent protein catabolic process, development, and ATP synthesis-coupled proton transport. When categorized according to molecular function, 273 genes have enriched terms; these functions include ATP binding, catalytic activity, oxidoreductase activity, actin binding, lipid binding, and electron carrier activity. Additionally, 271 co-expressed genes (34.7%) were enriched in 20 KEGG pathways including metabolic pathways, oxidative phosphorylation, focal adhesion, tight junction, and PPAR signaling pathway. The PPAR signaling pathway is a nuclear hormone receptor containing pathway that plays a role in lipid metabolism. Genes involved in PPAR signaling pathway were associated with the intramuscular fat content^118^. Our results showed that most of the enriched GO terms belonged to lipid metabolism, energy production and conversion, and protein posttranslational modification and turnover. Genes with similar annotations have been previously reported to contribute to muscle growth and quality traits in rainbow trout^14,73–75^. Thus, DE lncRNAs may contribute significantly to muscle growth and thereby impact muscle characteristics through their interaction with genes affecting muscle food quality traits. Furthermore, the results support the regulatory mechanism of lncRNAs through mediation of cellular energy responses^119^.

### Transcription factor binding sites (TFBS) in promoter regions of DE lncRNAs and their co-expressed protein-coding genes

In this study, we scanned 500 nts upstream of 229 DE lncRNAs and their co-expressed protein-coding genes to predict TFBS in their promoter regions. Co-expressed genes, exhibiting similar expression patterns, are expected to be controlled by similar regulatory mechanisms and are likely regulated by the same transcription factors^120,121^. A total of 26 binding motifs that have a role in skeletal muscle development were used for scanning the promoter region of co-expressed protein-coding and noncoding genes. A total of 209 (91.3%) DE lncRNAs and 946 (91.8%) co-expressed, protein-coding genes harboring the same TFBS have been identified. Seventy-one DE lncRNAs and 340 co-expressed protein-coding genes had putative TFBS for myogenin, while 52 DE lncRNAs and 246 co-expressed, protein-coding genes had putative TFBS for myoD gene. Myogenin and myoD control determination and terminal differentiation of skeletal muscle cells. Supplementary file Table S12 contains all pairs of co-expressed genes and their common TFBS. Previous studies focused on the pivotal role of miRNAs in the regulation of major myogenic pathways^122^. The current results propose a potential post-transcriptional regulatory role for lncRNAs in myogenesis.

## Conclusion

Muscle yield and quality traits are determinants of the aquaculture industry profitability and consumers’ satisfaction. These traits result from multifactorial interactions and given that the largest part of the transcriptome is noncoding and the role of lncRNA in regulating myogenesis is increasing, we sought to perform an integrated analysis of mRNA and lncRNA in fish families showing divergent phenotypes for muscle yield and quality traits. We identified some candidate protein-coding genes that were DE in families of contrasting phenotypes. Of them, *MYSS* and *TNNI2* isoforms, and *STC* were explaining part of the phenotypic variations, suggesting them as potential markers for WBW and muscle yield, and shear force, respectively. However, the lncRNA showed higher variability in terms of expression between divergent families. Given the fact that lncRNAs are poorly conserved, we identified networks/hubs between DE lncRNAs and their overlapping, neighboring, or distantly located on the genome based on expression correlation analysis. For example, the overlapping Omy500041161/*LIPL* and Omy400178299/*TGF-β* gene pairs revealed significant association with fat content and WBW, respectively. Additionally, lncRNA (Omy500089619) exhibited significant correlations with WBW, muscle yield, and fat content. These genes are good candidates for future knockdown studies to verify their roles in controlling the phenotypic variations. This study revealed complex microRNA sponge effects for lncRNA (Table S10) that may contribute to fast/efficient growth rates by controlling genes belonging to protein catabolic/anabolic pathways. Further, promoter regions of DE lncRNAs and their co-expressed protein-coding genes harbored similar TFBS necessary for muscle development, suggesting common transcription initiation mechanisms. Therefore, the current study highlights the possible regulatory interactions exerted by noncoding RNAs to control expression of protein-coding genes that impact muscle quality traits and adds additional layers of complexity that may help in understanding the molecular network of muscle development.

## Electronic supplementary material

Supplementary Dataset
